# A minimum dataset for destination therapy with left ventricular assist device: the evidence that matters to decision makers

**DOI:** 10.1017/S0266462325000054

**Published:** 2025-01-17

**Authors:** Janet Puñal-Riobóo, Maria José Faraldo Vallés, Natalia Nogueira Uzal, Hannah Patrick, Leonor Varela-Lema

**Affiliations:** 1 Galician Health Knowledge Agency (Avalia-t ACIS), Santiago de Compostela, Spain; 2 Pharmacist Public Health Inspector, Galician Department of Health, Santiago de Compostela, Spain; 3 National Institute for Care and Health Excellence (NICE), London, UK; 4 Preventive Medicine and Public Health Department, University of Santiago de Compostela, Santiago de Compostela, Spain

**Keywords:** heart-assist devices, destination therapy, minimum dataset, clinical decision-making, evidence gaps and real-world data

## Abstract

**Background:**

Left ventricular assist devices (LVAD) are a therapeutic option in patients with advanced heart failure (HF) not a candidate for cardiac transplant as destination therapy (DT). However, important uncertainties remain regarding the use of LVAD in the long-term in real practice settings. When planning registries, it is important to choose the appropriate outcomes that ensure comparability and reduce the possibility of bias.

**Aim:**

The purpose of this study was to establish a minimum dataset (MDS) that should be collected in all LVAD for DT registries to meet the needs and demands of Health Technology Assessment (HTA) doers and health professionals.

**Methods:**

To design the MDS for LVAD, a preliminary list of outcome domains and data items were developed attending to the gaps and research needs derived from existing evidence coming from HTA carried out at the European Network of Health Technology Assessment (EUnetHTA) level. The list of data items and domains was agreed upon by all involved HTA organizations and a three-round Delphi was conducted among an experienced panel of cardiologists to rate the importance of the items for measuring uncertainty gaps.

**Results:**

After the three-round Delphi process, the expert panel reached a consensus regarding eighteen outcomes and forty-seven variables divided into seven main domains (safety, effectiveness, patient acceptability, satisfaction, healthcare system impact, pharmaceutical management, and technique-related factors).

**Conclusions:**

The MDS of outcomes and measures, developed based on research gaps and needs, can allow for standardizing data collection and improving the quality of data for decision making and practice.

## Background

Heart failure (HF) is a global pandemic affecting an estimated 64 million people worldwide (1). Despite the progress made during the last twenty years in the medical treatment of HF, the percentage of patients in whom the disease progresses to an advanced or terminal stage remains high. When medical therapy ceases to be effective, cardiac transplantation is considered the treatment of choice, although this is limited by the availability of organs. In this context, left ventricular assist device (LVADs) are commonly used as a bridge to transplant therapy until a compatible donor is available (2).

However, many patients who are elderly or have multiple co-morbidities are not candidates for cardiac transplant and require implantation of LVAD as DT. The long-term functionality and safety outcomes are also encouraging the use of these implantable devices as DT when organ donors are not available. During the last decade, the use of these devices as DT has steadily increased but key issues exist surrounding the selection of suitable patients that would most benefit from the implantation of these devices in real practice (3;4). Uncertainties remain regarding device-related complications, patient-reported outcomes (quality of life, satisfaction) and management of patients to optimize outcomes. Randomized controlled trials (RCT) remain the gold standard for assessing effectiveness and safety but are deemed inappropriate for this purpose because they enroll a highly selective population that in many cases differs from the use in real-world conditions (5;6). Several studies suggest that there are discrepancies between the hard outcomes and the patient-reported outcomes, with the devices performing worse in real-life conditions than in randomized controlled trials (7).

Patient registries constitute an alternative methodology for real-world data (RWD) gathering. In several countries, LVAD registries have been mandated by healthcare bodies post-approval to monitor outcomes and support coverage decisions (8;9). Professional associations, like the European Association for Cardiothoracic Surgery, are also running registries to collect information on patients receiving mechanical circulatory support to support research (10). Some of these registries show that mortality in LVAD patients is high, and complications are common (8), but comparisons between studies are difficult because of the differences in type of devices, patient selection criteria, outcome definition, and outcome reporting. Patient registries have been commonly criticized because they tend to lack standardization in data collection and have a poor reporting of outcome results, leading to outcome-related bias in these studies (11). This undermines the generalizability and the utility of Health Technology Assessment (HTA) and decision making.

The development of a consensus-based agreed minimum dataset (MDS) collection could contribute to overcoming these problems (12;13). MDSs have been defined as a coherent set of data elements that should be collected for specific categories or domains of healthcare (14). The development of MDSs for LVAD DT registries could facilitate standardized care and ensure appropriate evidence is generated for informing decision making and practice (15). The existence of MDS would also facilitate cross-border collaboration on the generation and exchange of RWD not only on clinical aspects but also on organizational, ethical, social, and legal aspects that can determine its use in an NHS. This could be especially relevant considering the few patients that might benefit from LVAD DT implantation (16).

The purpose of this study was to establish a list of data elements that should be collected in all LVAD registries to meet the needs and demands of HTA doers and health professionals. This work was conducted as part of the European Network of Health Technology Assessment (EUnetHTA) Joint Action 3 Work Package (WP) 5 Strand B activities, whose general aim was to help in generating optimal and robust evidence for health technologies (pharmaceuticals or others) throughout the technology lifecycle, bringing benefits for patient access and public health (17).

## Methods

### Study steps

**Figure 1.**
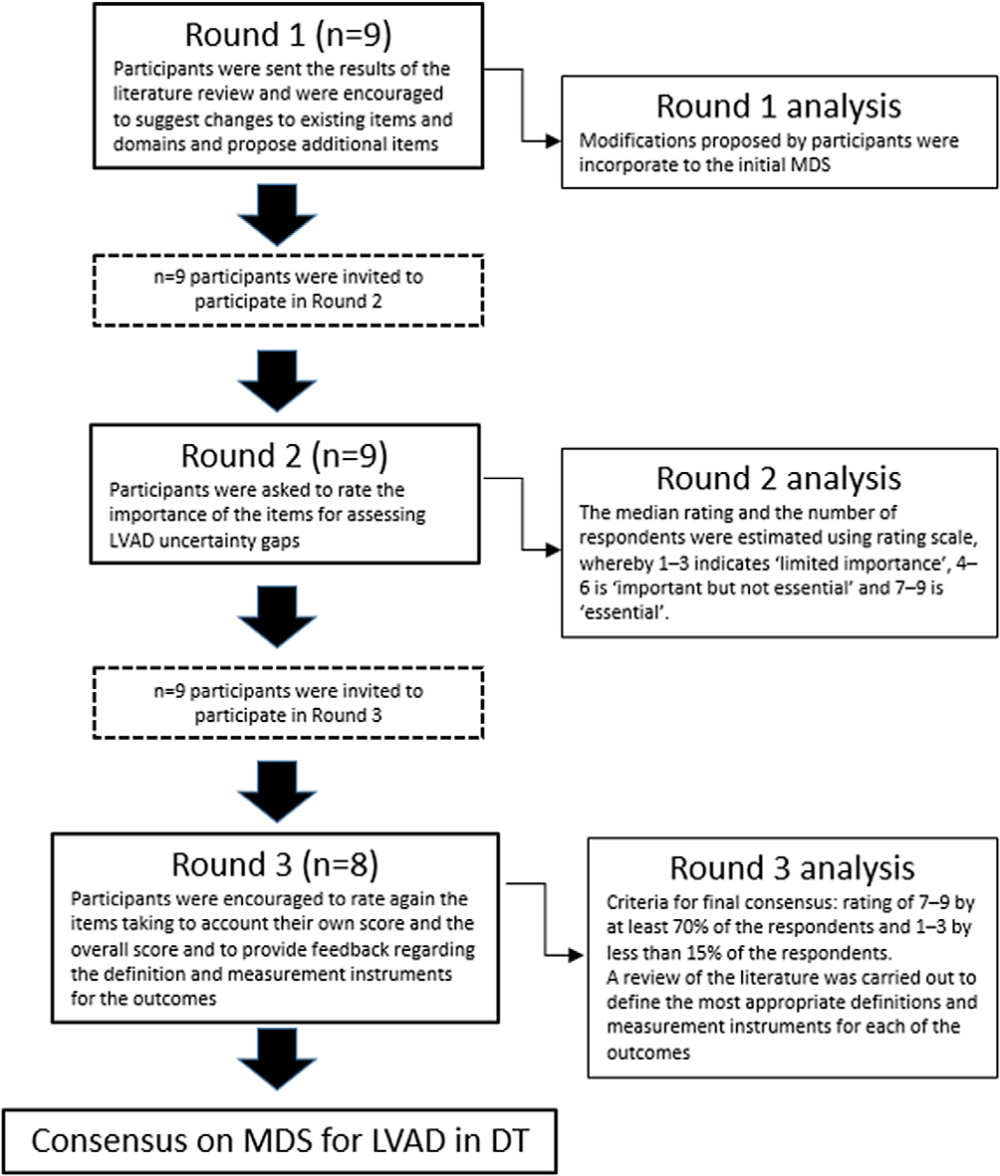
Steps followed for the development of MDS for LVAD in DT.

The MDS was developed following a four-step approach (Figure 1): 1) Identification of common uncertainties/gaps, 2) Development of the preliminary list of core domains and data elements to be collected in the registry, 3) Definition of MDS to be collected in routine practice 4) Elaboration of measurement instruments.

The MDS was developed by experienced HTA doers from three EUnetHTA organizations who had been involved in the development of HTA reports on LVAD as DT: Galician Health Knowledge Assessment Agency (ACIS), Haute Autorité de Santé (HAS) and National Institute for Health and Care Excellence (NICE). The conduct and reporting of this MDS adhere to the framework proposed by Svensson-Ranallo et al (14), except for patient involvement which was not feasible due to the very early adoption of these devices when the MDS was developed, further hindered by the fact that there are very few patients candidates for DT and their health status is commonly very compromised (18). The conduct and reporting of Delphi studies follow the methodological considerations or reporting for studies using the Delphi technique to determine which outcomes or domains to measure in clinical research studies provided by Sinha and colleagues (19).

### Identification of common uncertainties/gaps

A systematic review of HTA reports from European countries on LVAD as destination therapy was conducted. Four LVAD HTA assessments were identified (Spain (20), UK (21), Italy (22), and Belgium (23)). Evidence gaps and research needs were derived from existing evidence coming from these assessments.

The preliminary list of research gaps/needs was developed attending to the issues where no studies were identified, where there was insufficient information or the quality of the studies was low. Four EUnetHTA partners and organizations that had produced the LVAD HTA assessments were contacted for confirmation and clarifications with regard to identified evidence gaps/research needs. The identification of gaps and formulation of research recommendations was done in accordance with the EUnetHTA position paper on how to best formulate research recommendations for primary research arising from the HTA (24).

## Development of MDS outcomes and variables

The preliminary MDS was developed by the research team based on the PICOS characterization of the research gaps. The outcomes (i.e., the group of variables that asses the same issue) and variables (i.e., each item of a given outcome) that make up this MDS were then grouped in domains and shared once more with the four organizations involved in the HTAs for comments. All these organizations made contributions to the list and agreed on the final MDS.

## Definition of MDS domains

Using the Delphi technique, a multi-round online Delphi survey was performed to obtain consensus among clinical experts regarding the importance of these MDS for measuring LVAD existing uncertainty gaps. Given the complexity of the procedure, these experts were purposely selected according to their experience in LVAD implantation. These experts were mainly identified through the Spanish Society of Cardiology and European Society of Cardiology. An invitation letter was sent to these Societies to identify suitable experts and these were contacted afterwards. All of the experts who agreed to collaborate signed the Declaration of Interest and Confidentiality Undertaking (DOICU) form by email. Although we relied on several associations of cardiac patients to identify patients, we could not find suitable candidates to collaborate in the Delphi.

Eight clinical experts coming from Spain and 1 from the UK agreed to participate in the Delphi survey. The 66.7 percent are male (*n* = 6). All were experienced cardiologists (>10 years) who were directly involved in the treatment or management of patients with end-stage heart failure. Of these, 33 percent were cardiovascular surgeons and the remaining 66 percent were cardiologists. Five of them were heads or coordinators of their units and two were representatives of the Spanish and European Society of Cardiology, therefore who are considered leaders in their field. The overrepresentation of cardiologists was intentional given that they are more intimately engaged with patients and cares and can therefore provide deeper insights into their perspectives and experiences.

### Round-1 Delphi

During round one participants were sent the results of the literature review, and the variable list and were asked to review this list and were encouraged to suggest changes to existing variables and domains and propose additional variables. The participants were presented with an Excel file with multiple working sheets. Each participant remained anonymous during the Delphi process.

### Round 2-Delphi

Participants were asked to rate the importance of the variables attending to the acceptability, feasibility, and appropriateness of the measures for assessing LVAD uncertainty gaps. The rating was performed using a modified version of the Grading of Recommendations Assessment, Development, and Evaluation rating scale, whereby 1–3 indicates ‘limited importance’, 4–6 is ‘important but not essential’ and 7–9 is ‘essential. Variables that reached a median score ≥ 7 with consensus (≤2 panelists rating with a score out of range that contains the median) were considered for the final list and were not included in the next round. Those variables rated with a median score ≤ 3 with consensus were considered of limited importance and were disregarded. And variables rated with a median score 4–6 or ≥ 7 but with no consensus were included in round 3.

### Round 3-Delphi

In the third round participants were provided feedback regarding the comments received, their own score, and the overall score (median score) for each of the variables rated in the previous round and were given the opportunity to modify their score in view of the comments and overall rating. Criteria for final consensus were defined a priori as a rating of score ≥ 7 with consensus.

### Elaboration of measurement instruments

Once a consensus was reached on the MDS, a specific bibliographic review of the literature was carried out to define the most appropriate definitions and measurement instruments (a score or checklist recommended to asses a given outcome) for each of the outcomes. These definitions were once again sent to the clinical experts for corrections and comments.

## Results

The analysis of research gaps/needs generated an initial list of seventy variables relating to eighteen outcomes which were grouped into seven domains: baseline patients’ characteristics (*n* = 21) (including comorbidities and cardiovascular history), technique-related factors (device trademark, availability of transplant unit in the center) (*n* = 2), pharmacological management (*n* = 2), safety (n = 21), effectiveness (*n* = 14), satisfaction and acceptability of the patient (*n* = 2) and cost-effectiveness, budget impact and organizational impact (*n* = 8).

### Round-1 Delphi

Participants proposed minor modifications to the naming of five variables and a major change to one variable (LVEF, left ventricular ejection fraction for end-diastolic volume). They proposed adding four new variables (chronic right-sided heart failure, acute endocarditis, aortic regurgitation grade, and learning curve). The preliminary list of variables (*n* = 74) and domains is shown in Supplementary Table 1.

### Round-2 Delphi

**Figure 2.**
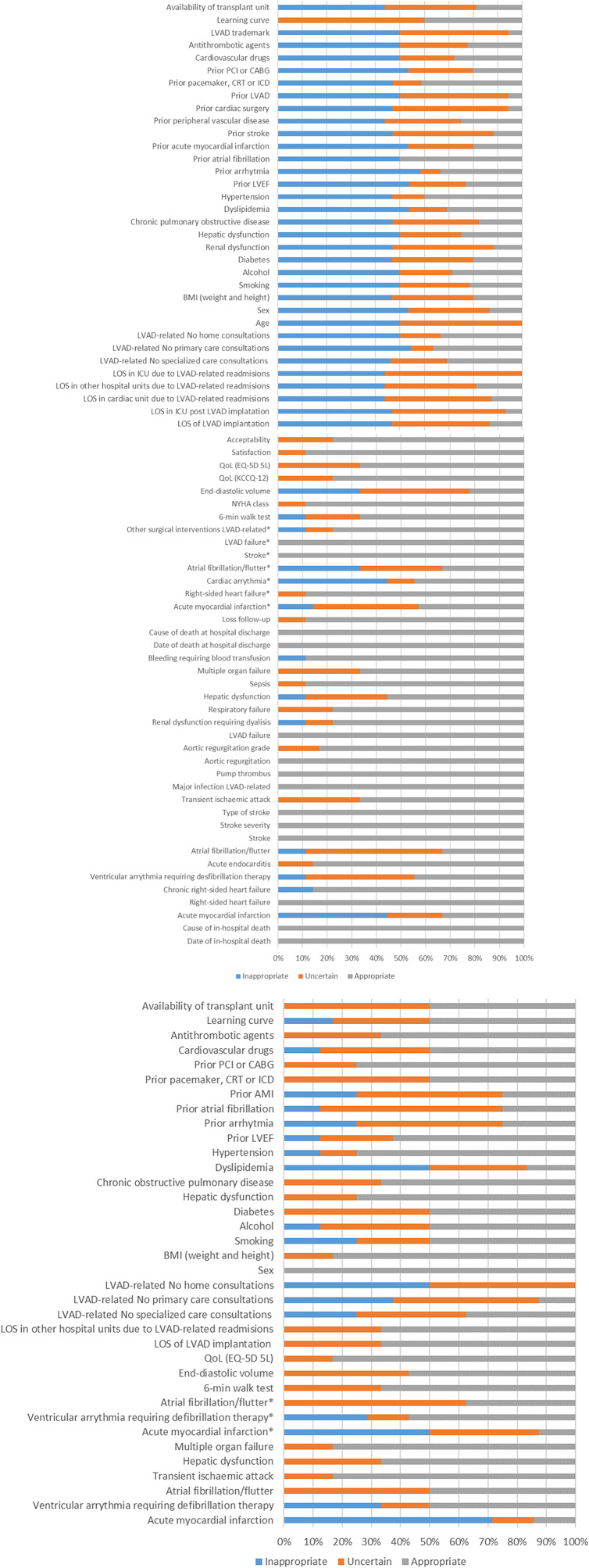
All variables ratings in round 2 (a) and 3 (b). a) Panelist ratings distribution in round 2 of Delphi consensus. *These variables were used to calculate event-free survival after LVAD implantation. Abbreviations: EQ-5D 5 L, Euro Quality of Life-5 dimensions 5 levels; KCCQ, Kansas City Cardiomyopathy Questionnaire; LVAD, left ventricular assist device; LVEF, left ventricular ejection fraction; NYHA, New York Heart Association; QoL, quality of life. (b) Panelist ratings distribution in the round 3 of Delphi consensus. *These variables were used to calculate event-free survival after LVAD implantation. Abbreviations: EQ-5D 5 L, Euro Quality of Life-5 dimensions 5 levels; KCCQ, Kansas City Cardiomyopathy Questionnaire; LVAD, left ventricular assist device; LVEF, left ventricular ejection fraction; NYHA, New York Heart Association; QoL, quality of life.

All of the participants answered the questionnaire (99 percent rated all the questions; two failed to rate one variable). Out of the seventy-four variables, thirty-seven variables obtained a median score ≥7 with consensus reached and were not included in the third round (Figure 2).

### Round-3 Delphi

The fulfillment rate of sheets by panelists was 85.8 percent during round 3. In round 3 of the Delphi a total of thirty-seven variables were scored again by panelists who knew their own and overall ratings.

During this third phase, ten of the remaining variables were rated as essential (median score ≥ 7) with the consensus reached (figure 2).

The final MDS proposal is composed of eighteen outcomes and forty-seven variables divided into seven domains.

## Measurement instruments

Table 1 shows the final list of items and measurement instruments. Participants agreed that data on safety, effectiveness, and health system impact should be collected at hospital discharge and at least 1 month, 3, 6, 9, 12, 18, 24 months, and once a year afterwards.

**Table 1.** Final list of items (outcomes and variables) and measurement instruments classified by domains

Domain	Outcomes	Measurement instrument/definition	Follow-up
**Baseline patients’ characteristics**	**Variables related to the patient:** age, sex, BMI, renal dysfunction, hepatic dysfunction, hypertension	-ACC clinical data standards -ICHOM *modified	*Pre-LVAD implantation*
	**Cardiovascular history**: prior stroke, PCI or CABG, cardiac surgery or LVAD.	EACTS Adult Cardiac Database, Version 2.0 -American College of Cardiology	
**Pharmacological management**	Antithrombotic drugs	-ATC WHO 2019 Guidelines	
**Factors related to the technique**	Device trademark	Not applicable	
**Safety**	**In-hospital death:** date and cause of death	-ACC Clinical Data Standards -INTERMACS Adverse Event Definition -ACC Clinical Data Standards	*Perioperative LVAD implantation*
	**Cardiac adverse events**: acute endocarditis, right-sided heart failure, chronic right-sided heart failure		*Post-LVAD implantation*
	**Neurological adverse events**: stroke (yes/no, type and severity), transient ischaemic attack	-ACC Clinical Data Standards -INTERMACS Adverse Event Definition -CDISC. Standardized Definitions for CV and Stroke end point events in clinical trials (Karen A. Hicks, 2014)	
	**Other serious adverse events:** renal dysfunction, respiratory failure, hepatic dysfunction, sepsis, bleeding requiring blood transfusion, and multiple organ failure	-INTERMACS Adverse Event Definition The Third International Consensus Definitions for Sepsis and Septic Shock (Sepsis–3) EACTS Adult Cardiac Database,Version 2.0 Martin B. Leon, 2011 GUSTO definition	
	**LVAD device-related adverse event:** major infection LVAD-related, pump thrombus, aortic regurgitation (yes/no and grade), LVAD major failure	-Zoghbi, WA 2017. Valvular Regurgitation -INTERMACS Adverse Event Definition	
**Effectiveness**	**Overall survival**: date/cause of death and loss follow-up	ACC Clinical Data Standards	*Post-LVAD implantation*
	**Survival free of events**: right-sided heart failure, stroke, LVAD replacement or explant, other surgical interventions LVAD-related	Not applicable	
	**Functional capacity:** 6-min walk test (6 MWT), NYHA class.	-New York Heart Association -American College of Cardiology -ATS Statement-Guidelines for the 6-MWT	
	**Quality of life**: Kansas City Cardiomyopathy Questionnaire (KCCQ–12), EuroQol–5D (EQ–5D)	-KCCQ-short version -EuroQoL 5D–5L version	
**Patient or caregiver acceptability or satisfaction**		Adaptation of the SATISCORE patient satisfaction questionnaire for cardiac surgery (Spanish)^a^	*Post-LVAD implantation*
**Health system impact**	LOS in ICU post LVAD implantation	Not applicable	*Post-LVAD implantation*
	LOS in ICU due to LVAD-related readmissions		
	LOS in cardiac unit due to LVAD-related readmissions		

Abbreviations: BMI, body mass index; CABG, coronary artery by-pass graft; CV, cardiovascular; ICD, implantable cardioverter-defibrillator; ICU, intensive care unit; LOS, length of hospital stay; LVAD, left ventricular assist device; LVEF, left ventricular ejection fraction; CRT, cardiac resynchronization therapy; PCI, percutaneous coronary intervention.

a The following issues are proposed to measure the satisfaction and acceptability of the patient/caregiver (5-points Likert scale: 1 = very dissatisfied; 2 = dissatisfied; 3 = neither satisfied nor dissatisfied; 4 = satisfied; 5 = very satisfied): (a) In general, how satisfied are you living with LVAD and (b) Indicate the degree of agreement with the following statement: “If I found myself the same as before, I would have surgery again.”

## Discussion

Many concerns have been identified in different registries from across diverse settings including, among others: heterogeneity in the patient selection, lack of transparency in outcome selection and reporting, and poorly defined outcomes. These concerns seem to limit the data utilization for decision making and also impedes the performing of pooled analysis (25). In this scenario, the development and implementation of an MDS could improve the consistency and transparency in outcomes reporting. Moreover, the standardization of outcomes could increase the possibility of grouping results and performing comparative analysis between different strategies of treatment of a given disease that is considered essential in the decision-making process.

The current MDS proposal for LVAD as DT includes a set of key data elements for monitoring existing evidence gaps of LVAD, which are viewed as feasible to collect in real clinical practice, that is, outside of clinical trials. The major value of this MDS proposal resides in that it has been developed based on previously agreed-upon evidence gaps and research needs identified by different HTAs and prioritized by clinicians. HTAs are acknowledged to be a source of systematically generated, comprehensive information for formulating researchable questions that are relevant to decision makers (26;27). However, relying solely on the producers of HTA reports to identify research gaps might result in an extensive list of items, but not necessarily the most relevant ones to clinicians or patients (28). The involvement of stakeholders, especially clinicians and patients ensures that the needs of end users are met and also, that the data is feasible to collect in real-world practice.

In our study, we did not achieve the participation of individual patients or representatives in our panel. This was probably due to the fact that there are very few patients with end-stage heart failure who are ineligible for heart transplantation and these commonly have a poor health status. However, we are confident that our LVAD MDS could be aligned with the patients’ perspectives. We did not observe significant differences in the judgment of patient-centered outcomes in our study with respect to the ICHOM standard pragmatic patient-centred outcome set on heart failure patients (29) aimed to improve patient care and permit comparison across regions and healthcare systems. This outcome set was composed of seventeen items related to survival (mortality), functional (symptoms control, living independently, and so forth assessed by New York Heart Association-NYHA class or Kansas City Cardiomyopathy Questionnaire-KCCQ), psychosocial (Quality of Life-QoL, depression, anxiety, etc.) or burden of care (a complication of treatment, number of hospital readmission, length of stay, etc.). In addition, they provided a set of adjustment variables in order to allow the comparability between regions and healthcare systems (29). Our MDS covers all these items except for the two patient-centered outcomes related to psychosocial status (i.e., depression and anxiety). Instead, the MDS includes the EuroQoL-5D-5L QoL scale which comprises four other dimensions besides anxiety and depression. This scale is the one most commonly used for the estimation of health utility and quality-adjusted life-years (QALYs), which is essential for cost-utility analysis (CUA) and economic evaluations (30).

Despite the rapidly growing number of LVAD implants, there are limited and contradictory data about patients’ device acceptance and no data about the relationship between patients’ device acceptance and the psychological well-being and QoL of LVAD recipients. To account for these uncertainties, alongside commonly used patient-reported outcomes (PROs), like quality of life (evaluated by EuroQoL-5D-5L and KCCQ-12 questionnaires), we included two broad questions on acceptability and satisfaction which were adapted from previously validated patient-based questionnaire developed for evaluating patient and carers satisfaction after cardiac surgery. Although in the future these questions could be more streamlined, we are confident that they will allow for assessing participants’ global well-being and satisfaction with their lives.

In the same way, as Burns et al (29), we established stratification factors (i.e., by device type, by the availability of transplant unit, and by baseline characteristics of patients) of variables that could allow a stratified analysis of clinical trial’s results and even comparison of studies conducted in different health systems or cardiac patient populations. Baseline characteristics of patients, such as comorbidities or prior cardiac or coronary surgeries are liable to modify the safety or efficacy results of a given health intervention. Therefore, a *post hoc* analysis of outcomes throughout these stratification factors could be very helpful to improve the identification of the best heart failure candidate for whom clinical results would be optimal (31;32).

The final MDS developed by our group includes the core outcomes of mortality, quality of life, hospitalization, and cerebrovascular complications that were established in the “COS Adult Cardiac Surgery” (33). In relation to mortality, the list not only includes survival but also event-free survival (including events such as right heart failure, stroke, LVAD replacement or explant, and other surgical interventions (LVAD-related) as these complications are directly related to hospital readmissions, reinterventions, and finally with QoL of heart failure patients. The MDS list also includes complications specifically related to the LVAD device, including, among others, device failure, bleeding, infection, and stroke. These safety outcomes were also considered primary end points in other clinical trial proposals, as these are viewed as critical from a regulatory perspective, due to the high risk of hospital readmission and cost associated with these devices (34).

Finally, our proposal included a definition and follow-up for each variable, reviewed and agreed upon by participants in the Delphi consensus, which could facilitate the implementation of our MDS in different health systems or settings for informing the decision-making process or even develop clinical trials.

The Delphi process is a widely used method for achieving consensus among experts on the development of minimum data elements by means of an iterative, structured, and transparent process. As such, it is commonly used in the development of COS (35). Two examples of COSs based on COMET methodology are performed on cardiac patients although these aimed at patients with coronary artery disease treated with cardiac surgery (33) and patients who suffered a cardiac arrest (36). In the COSs for cardiac arrest, Haywood et al (36) employed a two-round Delphi study and a 2-day meeting in small groups of discussions; after that, they developed a core measurement/variable set aligned to the core domain set. However, Benstoem et al (33) used a three-round online Delphi survey.

Both authors concluded that the COMET methodology enhances the consistency, transparency, relevance, and accuracy of a given COSs in a specific area. Moreover, the participation of multiple stakeholders and the application of an agreed methodology during the COSs development could assure its applicability and implementation in clinical trials limiting the reporting bias and heterogeneity across these. As Benstoem et al (33) highlighted the next step during the COSs development process, is to identify the core measures aligned to the core domain set. In our study, we elaborated a MDS through a three-round Delphi consensus of clinical experts of different specialties following a robust methodology proposed by the COMET initiative. Therefore, we expect that the MDS proposed could have great relevance for LVAD registries but the considerations could also be applicable to clinical trials or observational trials.

The current study has some strengths and limitations. We consider that the recruitment of multidisciplinary independent experts from different specialties with experience in LVAD and recognized leadership in the field is a key strength of the study. Although we relied on the opinion of a small number of experts, it has been previously demonstrated that reliable results can be obtained with small expert panels selected upon strict criteria (22). A potential limitation concerning the experts involved in the study is that the majority are from Spain, with only one expert from the United Kingdom, potentially affecting the generalizability of the study’s findings to other countries. We consider that the scorings are unlikely to be influenced by country-specific practices, as the experts involved are prominent leaders in their fields, often serving as heads of their units or representatives of key scientific societies, providing them with extensive knowledge of current best practices. However, as with many clinical matters, individual beliefs may be shaped by personal experiences and perceptions regarding the feasibility and utility of specific data. These perceptions could also be influenced by factors such as clinical specialties (e.g., surgeons vs. cardiologists) or local policy and contextual considerations.

Although no standardized recommendations exist regarding the stakeholders to be involved in such processes it is widely acknowledged that the stakeholder group should include key experts with experience in the investigation, management, or conduct of studies in the target population (37). We consider that the inclusion of clinician’s with experience in LVAD was particularly critical for our study, given the complexity of the LVAD procedure and the significant evidence gaps related to device-specific outcomes. The inclusion of clinicians’ leaders in the field was also essential to ensure the dataset’s feasibility for implementation in real practice, which was one of our primary objectives. However, it cannot be dismissed that including other stakeholders with different expertise, such as decision makers or HTA doers could have provided additional valuable perspectives and widened the generalizability (38).

Another potential limitation of the current MDS relates to the lack of inclusion of patients or carers. Although confident that the MDS covers participants’ global well-being and satisfaction with their lives, a risk exists that it may not encompass all patient-relevant outcomes.

Nonetheless, we consider that these potential biases do not undermine the value of the study as the LVAD MDS represents the first standardized framework for data collection in this field, which could be adapted and expanded upon with additional data elements as required. Although multicentre registries from different countries showed different LVAD DT implant rates (9;16), the main uncertainties or evidence gaps identified from evidence do not differ in different settings (39). Then, the clinical relevance of the variables proposed should not be affected by the number of patients who are candidates for LVAD as destination therapy. In fact, it could be of greater interest to perform registries based on MDS, as the one we proposed in our work, in those settings with a high level of use of LVAD as destination therapy due to adverse events associated with their use.

Our MDS is very valuable in the sense that it covers most of the relevant gaps identified by HTA doers in relation to LVAD in DT incorporating a wide array of variables pertaining to safety, effectiveness, and organizational aspects, even if they are not always directly related to patients.

In conclusion, we have developed a minimum set of outcomes and variables that could enhance the use of LVAD registries for decision making and clinical practice. The methodology used for elaborating our dataset, based on evidence gaps collected by HTA assessments and a Delphi consensus, constitutes an innovative approach that can allow for improving the quality of data and standardizing data collection. This last issue could also be ensured by the use of recognized measurement instruments/definitions that have been previously developed by the most relevant scientific societies. The MDS is currently being applied in the Spanish prospective LVAD Registry implemented at the National Health Care system to assess acceptability. We recommend that these dataset be also implemented in other registries or trials implemented in other countries as part of their HTA decision-making process. Broad implementation is critical, but can only be achieved by raising awareness, especially at the HTA or policy making level, regarding the importance of harmonizing high-quality data collection, particularly for rare events or indications. This could contribute to reducing the variability observed in the reporting of outcomes and increase the possibility of data pooling. Moreover, the implementation of our proposal, based upon agreed evidence gaps, could provide additional data addressing uncertainties related to organizational and cost-effectiveness issues.

## Supporting information

Puñal-Riobóo et al. supplementary material Puñal-Riobóo et al. supplementary material

## Supplementary material

The supplementary material for this article can be found at http://doi.org/10.1017/S0266462325000054.
