# Supplementary material for: A minimum dataset for destination therapy with left ventricular assist device: the evidence that matters to decision makers
Source: Int J Technol Assess Health Care. 2025 Jan 17;41(1):e8. doi: 10.1017/S0266462325000054 (PMC11811952; doi:10.1017/S0266462325000054)
Supplement: Puñal-Riobóo et al. supplementary material [file S0266462325000054sup001.docx]

Table 1. Preliminary core outcome and variable set classified by main domains

| **Domain** | **Outcome** | **Variable** |
| --- | --- | --- |
| **Baseline patients’ characteristics** | - Variables related to the patient/comorbidities | Age, sex, BMI, smoking, alcohol, diabetes, renal dysfunction, hepatic dysfunction, chronic pulmonary obstructive disease, dyslipidemia, hipertension |
|  | - Cardiovascular history | Prior LVEF, arrhythmia, atrial fibrillation, acute myocardial infarction, stroke, peripheral vascular disease, cardiac surgery, LVAD, pacemaker/CRT/ ICD, PCI or CABG |
| **Pharmacological management** | | - Cardiovascular and antithrombotic drugs |
| **Factors related to the technique** | | - Device trademark - Availability of transplant unit - Learning curve |
| **Safety** | - In-hospital death | - Date of death - Cause of death (CV and non-CV death) |
|  | - Cardiac adverse events | - Acute myocardial infarction - Right-sided heart failure - Chronic right-sided heart failure - Atrial fibrillation/flutter - Ventricular arrhythmia requiring defibrillation therapy - Acute endocarditis |
|  | - Neurological adverse events | - Stroke (type and severity) - Transient ischaemic attack |
|  | - Other serious adverse events | - Renal dysfunction requiring dialysis - Respiratory failure - Hepatic dysfunction - Sepsis - Bleeding requiring blood transfusion - Multiple organ failure |
|  | - LVAD device-related adverse event | - Major infection LVAD-related - Pump thrombus - Aortic regurgitation - Aortic regurgitation grade - LVAD major failure |
| **Effectiveness** | - Overall survival | - Date of death - Cause of death - Loss follow-up |
|  | - Survival free of events | - Date of acute myocardial infarction - Date of right heart failure - Date of ventricular arrhythmia requiring defibrillation therapy - Date of atrial fibrillation/flutter - Date of stroke - Date of LVAD replacement or explant - Date of other surgical interventions LVAD-related |
|  | - Functional capacity | - 6-min walk test (6 MWT) - NYHA class - End-diastolic volume |
|  | - Quality of life | - Kansas City Cardiomyopathy Questionnaire (KCCQ-12) - EuroQol-5D (EQ-5D) |
| **Patient or caregiver acceptability or satisfaction** | | Adaptation of the SATISCORE patient satisfaction questionnaire for cardiac surgery (Spanish)^1^ |
| **Health system impact** | - LOS for LVAD implantation | - Date of admission - Date of discharge |
|  | - LOS in ICU post-intervention | - Date of ICU admission - Date of ICU discharge |
|  | - LOS in cardiac unit readmission | - Date of cardiac unit readmission - Date of cardiac unit discharge |
|  | - LOS in ICU readmission | - Date of readmission in ICU - Date of ICU discharge |
|  | - LOS in other hospital unit readmission | - Date of other hospital unit readmission - Date of other hospital unit discharge |
|  | - LVAD-related specialized care consultations | - Number of specialized care consultations |
|  | - LVAD-related primary care consultations | - Number of primary care consultations |
|  | - LVAD-related home consultations | - Number of home consultations |

**Abbreviations:** BMI, body mass index; CV, cardiovascular; ICU, intensive care unit; LVAD, left ventricular assist device; LOS, length of hospital stay.

^1^The following issues are proposed to measure the satisfaction and acceptability of the patient / caregiver (5-points Likert scale: 1=very dissatisfied; 2=dissatisfied; 3=neither satisfied nor dissatisfied; 4=satisfied; 5=very satisfied): (a) In general, how satisfied are you living with LVAD and (b) Indicate the degree of agreement with the following statement: “If I found myself the same as before, I would have surgery again”.
